# Early aortic valve intervention in asymptomatic severe aortic stenosis: a clinical dilemma in evolution

**DOI:** 10.1093/ehjopen/oeaf114

**Published:** 2025-08-29

**Authors:** Patrizio Lancellotti, Augustin Coisne, Bernard Cosyns, Raluca Dulgheru, Madalina Garbi, Geu-Ru Hong, Jadranka Separovic Hanzevacki, Marco Moscarelli, Tadafumi Sugimoto, Erwan Donal, Khalil Fattouch, Gilbert Habib, Mani Vannan, Philippe Pibarot

**Affiliations:** GIGA Cardiovascular Sciences, Heart Valve Clinic, University of Liège Hospital, Avenue de l'Hôpital 1, 4000 Liège, Belgium; Department of Cardiology, University of Liège Hospital, Domaine Universitaire du Sart Tilman—B.35, Liège 4000, Belgium; Department of Cardiology, Univ. Lille, Inserm, CHU Lille, Institut Pasteur de Lille, U1011—EGID, Rue Calmette BP245, Lille 59019, France; Cardiovascular Research Foundation, 1700 Broadway, 9th Floor, New York, NY, USA; Centrum Voor Harten Vaatziekten (CHVZ), Vrije Universiteit Brussel (VUB), Universitair Ziekenhuis Brussel (UZ Brussel), Avenue du Laerbeek 101, 1090 Jette, Brussels, Belgium; In Vivo Cellular and Molecular Imaging (ICMI) Center, Vrije Universiteit Brussel (VUB), Avenue du Laerbeek 101, 1090 Jette, Brussels, Belgium; GIGA Cardiovascular Sciences, Heart Valve Clinic, University of Liège Hospital, Avenue de l'Hôpital 1, 4000 Liège, Belgium; Department of Cardiology, University of Liège Hospital, Domaine Universitaire du Sart Tilman—B.35, Liège 4000, Belgium; Royal Papworth Hospital, Cambridge University Health Partners, Papworth Road, Cambridge Biomedical Campus, Cambridge CB2 0AY, UK; Cardiology Division, Severance Cardiovascular Hospital, Yonsei University College of Medicine, 50-1, Yonsei-Ro, Seodaemun-gu, Seoul 03722, Republic of Korea; Department of Cardiovascular Diseases, University of Zagreb School of Medicine and University Hospital Centre Zagreb, Ulica Mije Kišpatića 12, 10000 Zagreb, Croatia; Department of Surgery & Cancer—Faculty of MedicineThe Faculty Building, Imperial College London, Exhibition Road, South Kensington, London SW7 2AZ, UK; Department of Cardiovascular Surgery, Maria Eleonora Hospital, GVM Care&Research, Viale della Regione Siciliana Nord Ovest, 1571, 90135 Palermo PA, Italy; Department of Cardiology, Nagoya City University Mirai Kousei Hospital, 2 Chome-1501 Sekobo, Meito Ward, Nagoya, Aichi 465-0055, Japan; Université de Rennes, CHU Rennes, Service de Cardiologie Inserm, LTSI-UMR 1099, 2 Rue Henri Le Guilloux, Rennes 35000, France; Department of Cardiovascular Surgery, Maria Eleonora Hospital, GVM Care&Research, Viale della Regione Siciliana Nord Ovest, 1571, 90135 Palermo PA, Italy; Cardiology Department, APHM, La Timone Hospital, Boulevard Jean Moulin, Marseille 13005, France; 2 Aix Marseille University, IRD, APHM, MEPHI, IHU-Méditerraneée Infection, 19-21 Boulevard Jean Moulin, Marseille 13005, France; Marcus Heart Valve Center, Piedmont Heart Institute, 95 Collier Rd NW Suite 5015, Atlanta, GA 30309, USA; Quebec Heart and Lung Institute, Université Laval, 2725 Ch Ste-Foy, Québec City, Quebec QC G1V 4G5, Canada

**Keywords:** Aortic stenosis, Risk stratification, Early intervention, Aortic valve replacement, Transcatheter aortic valve implantation

## Abstract

The management of asymptomatic severe aortic stenosis (AS) has traditionally relied on watchful waiting until the onset of symptoms or left ventricular dysfunction. However, the FDA’s approval of transcatheter aortic valve replacement (TAVR) with the Sapien 3 platform in May 2025, based on the EARLY TAVR trial, has intensified the debate over early intervention. This Viewpoint synthesizes evidence from randomized trials (RECOVERY, AVATAR, EVOLVED, EARLY TAVR) and registries (HAVEC, VALVENOR) to evaluate the role of early aortic valve replacement (AVR). Early intervention is associated with reductions in combined endpoints of cardiovascular hospitalizations, stroke, and mortality in selected patients, with the EARLY TAVR demonstrating a 50% reduction in major cardiovascular events. Nonetheless, evidence remains inconsistent, particularly in low-risk populations, as the EVOLVED trial showed no mortality benefit in patients with myocardial fibrosis, warranting cautious interpretation. A conservative surveillance strategy remains appropriate in some cases, supported by the low annual risk of sudden death (i.e. 0.65% per year) and ongoing concerns over valve durability and procedural risks. Given the heterogeneity of patient and valve phenotypes, a personalized risk assessment, combining clinical evaluation, biomarkers (troponin, BNP), and imaging (echocardiography, CMR), is proposed to identify high-risk patients and optimize the timing of early intervention. Expert heart team guidance is essential, and routine early intervention cannot yet be recommended. Further research is needed to refine strategies and improve outcomes in this evolving clinical landscape.

## Graphical Abstract

**Graphical Abstract:**
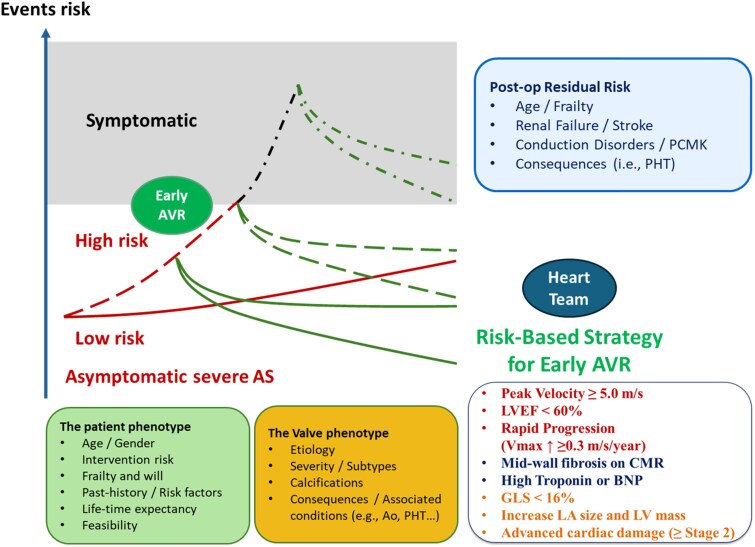

## Introduction

**Figure 1:**
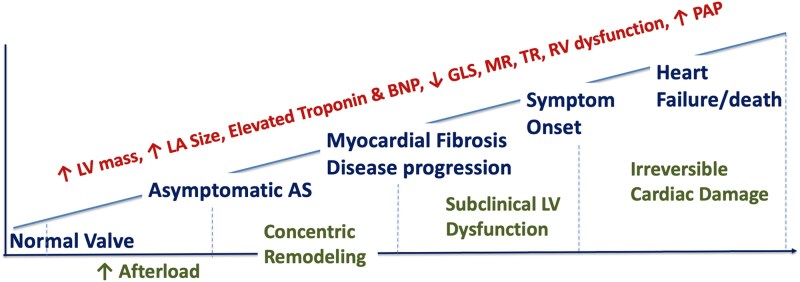
Pathophysiological cascade of aortic stenosis progression. This schematic illustrates the natural history of aortic stenosis from the normal valve state through asymptomatic phases, myocardial fibrosis, symptom onset, and eventual progression to heart failure or death. It emphasizes the silent nature of disease progression and the rationale for considering earlier intervention before irreversible damage occurs. AS, aortic stenosis; BNP, brain natriuretic peptide; GLS, global longitudinal strain; LA, left atrium; MR, mitral regurgitation; PAP, pulmonary arterial pressure; TR, tricuspid regurgitation.

In May 2025, the FDA approved the use of transcatheter aortic valve replacement (TAVR) with the Sapien 3 platform for patients with asymptomatic severe aortic stenosis (AS), based on the findings of the EARLY TAVR trial,^1^ marking a paradigm shift in aortic valve management (AVR). This progressive and ultimately life-threatening valvular heart disease primarily affects elderly individuals in western countries,^2^ with its prevalence projected to increase as the global population continues to age.^3^ The natural history of AS includes a prolonged asymptomatic phase marked by progressive myocardial/ventricular remodelling, impaired left ventricular (LV) strain, and biomarker elevation, setting the stage for myocardial fibrosis before symptoms emerge.^4–10^ This asymptomatic phase is typically followed by a steep increase in morbidity and mortality once symptoms such as angina, syncope, or heart failure develop.^11,12^ Subclinical myocardial changes—including concentric remodelling, impaired LV strain, and biomarker elevation—often develop silently during the asymptomatic phase, preceding overt symptoms (*Figure 1*).^13,14^ Traditionally, aortic valve replacement (AVR), whether surgical (SAVR) or TAVR, has been reserved for patients with overt symptoms or LV dysfunction (LVEF < 50%), consistent with historical evidence linking these conditions with poor prognosis.^15–17^ However, recent developments in imaging, biomarkers, and procedural safety have cast doubt on the wisdom of a symptom-based approach alone. The publication of new randomized controlled trials (RCTs) including RECOVERY,^18^ AVATAR,^19,20^ EVOLVED,^21^ and EARLY TAVR,^1^ as well as registry data, has reignited the debate regarding whether earlier intervention might yield better outcomes than the wait for symptom or clinical surveillance strategy.^6,8^

### The argument for early intervention

The argument for early AVR in asymptomatic severe AS stems from the growing recognition that a significant proportion of initially asymptomatic patients—over 30%—develop symptoms within the first year following diagnosis.^10,11,22^ Furthermore, increasing evidence indicates that pathologic cardiac remodelling often begins before the onset of symptoms,^13,14,22,23^ potentially leading to irreversible myocardial damage if intervention is delayed. Studies employing advanced imaging techniques, such as cardiac magnetic resonance (CMR) and strain echocardiography, have shown early signs of myocardial fibrosis and subclinical systolic dysfunction.^13,24^ Global longitudinal strain (GLS) has emerged as a powerful and sensitive marker of early myocardial dysfunction, often preceding declines in LVEF.^13,25–27^ In a large multicentre UK CMR study,^28^ myocardial fibrosis detected by late gadolinium enhancement was associated with markedly reduced survival after AVR, with no regression of scar burden post-intervention. This underscores the irreversible progression of structural damage once fibrosis develops, highlighting the critical importance of early intervention before scar formation.^29,30^ In this context, staging systems for asymptomatic AS have been proposed to better identify patients at risk of adverse outcomes, by integrating markers of early cardiac damage into clinical decision-making.^27^

**Figure 2:**
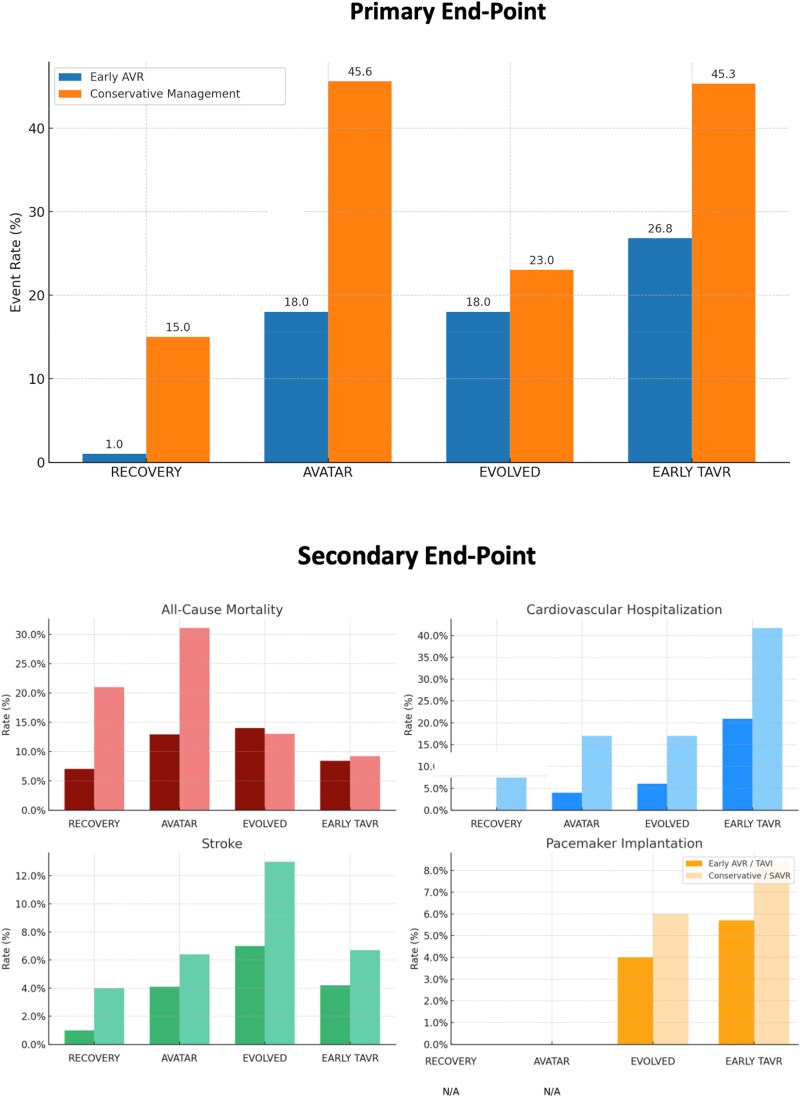
Clinical outcomes across randomized trials comparing early aortic valve replacement vs. conservative management. Bar charts show primary and secondary outcomes (all-cause mortality, cardiovascular hospitalization, stroke, and pacemaker implantation rates in randomized trials RECOVERY, AVATAR, EVOLVED, and EARLY TAVR). Outcomes are stratified by early AVR (dark bars) vs. conservative management (light bars). Pacemaker rates are shown only where available (N/A, not available). Data are from published trial reports.

Potential benefits of early intervention include reduced mortality, fewer cardiovascular rehospitalizations, improved quality of life, and prevention of disease progression and cardiac damage (e.g. myocardial fibrosis and systolic dysfunction).^31^ Clinical evidence further supports early intervention (*Figure 2*) (*Table 1*), though the small sizes and restrictive entry criteria limit their generalizability and have not yet influenced current guidelines. The RECOVERY trial (*n* = 145) enrolled patients with very severe asymptomatic AS (peak velocity ≥ 4.5 m/s or mean gradient ≥ 50 mmHg) and demonstrated a dramatic reduction in cardiovascular mortality with early SAVR compared to surveillance (1% vs. 15%; hazard ratio [HR], 0.09; 95% CI, 0.01–0.67).^18^

**Table 1:** Comparative outcomes in randomized controlled trials: early aortic valve intervention vs. conservative management

Study	Mean age	Primary endpoint	Mortality	Hospitalization	Stroke	Pacemaker	Interpretation
RECOVERY *N* = 72 vs. 73	64.2 years	Operative mortality, CV death 1% vs. 15%*	7% vs. 21%*	0% vs. 11%*	1% vs. 4%	N/A	Major mortality benefit with early SAVR
AVATAR *N* = 78 vs. 79	67 years	Death, MI, stroke, HFH 18% vs. 45.6%*	12.9% vs. 31.1%*	4% vs. 17%*	4.1% vs. 6.4%	N/A	Early SAVR reduces major events
EVOLVED *N* = 113 vs. 111	73 years	Death or AS-related hospitalization 18% vs. 23%	14% vs. 13%	6% vs. 17%	7% vs. 13%	4% vs. 6%	No mortality benefit, fewer emergency hospitalization and prevention of debilitating symptoms
EARLY TAVR *N* = 455 vs. 446	75.8 years	Death, stroke, CV hospitalization 26.8% vs. 45.3%*	8.4% vs. 9.2%	20.9% vs. 41.7%*	4.2% vs. 6.7%	5.7% vs. 8.4%	Reduced hospitalization and stroke; mortality difference not significant

CV, cardiovascular; MI, myocardial infarction; HFH, hospitalization for heart failure.

^*^ *P* < 0.05.

Similarly, the AVATAR trial (*n* = 157)^19,20^ showed that early SAVR significantly reduced a composite endpoint of all-cause mortality, stroke, myocardial infarction, or unplanned heart failure hospitalization compared to conservative management (18% vs. 45.6%; HR, 0.44; 95% CI, 0.23–0.85).^20^

The EVOLVED trial adopted a novel fibrosis-guided strategy, enrolling asymptomatic patients with severe AS and evidence of myocardial fibrosis on CMR (*n* = 224).^21^ Early AVR (SAVR or TAVR) led to a significant 63% lower risk of unplanned AS-related hospitalizations (HR, 0.37, 95% CI, 0.16–0.88) and better symptom status at 12 months, with more remaining in New York Heart Association (NYHA) class I. This reflects the design of the conservative arm, where intervention is delayed until symptoms or fibrosis progression necessitate it. Importantly, 45% of patients assigned to conservative management ultimately underwent TAVR compared to only 25% in the early AVR group. This suggests that delaying intervention often leads to urgent procedures following symptom onset, potentially exposing patients to higher procedural risks and poorer clinical conditions compared to planned elective AVR.

EARLY-TAVR randomized 901 asymptomatic severe AS patients to early transfemoral TAVR (*n* = 455) or clinical surveillance (*n* = 446).^1^ Early TAVR led to a 50% reduction in the composite endpoint of death, stroke, or unplanned cardiovascular hospitalization compared to surveillance (HR, 0.50; 95% CI, 0.40–0.63). The main benefit was a significant reduction in unplanned hospitalizations (20.9% vs. 41.7%; HR, 0.43; 95% CI, 0.33–0.55).^21^ However, the reduction in unplanned hospitalizations with early intervention is partly expected, as the conservative approach delays treatment until symptom onset, often resulting in hospitalization. Early TAVR also prevented rapid decline in quality of life and preserved better LV and atrial function. More than 30% of the patients in the clinical surveillance group presented with advanced symptoms before AVR regardless of the timing of conversion. Over 70% of patients initially assigned to clinical surveillance underwent AVR by two years, with 26% and 47% requiring intervention by 6 and 12 months, respectively. Alarmingly, nearly 40% of these patients presented with acute valve syndrome—including NYHA class III–IV heart failure, pulmonary oedema, syncope, ventricular arrhythmias, or resuscitated sudden cardiac death—and experienced a significantly higher 2-year rate of death, stroke, or hospitalization (14.9% vs. 6.8%) compared to the early TAVR group. Rehospitalization following TAVR is recognized as a powerful prognostic marker, strongly associated with increased mortality and substantial deterioration in health-related quality of life.^32^ Subgroup analysis of 798 patients showed that higher NT-proBNP and hs-cTnT levels were associated with worse outcomes. Notably, the benefit of early TAVR was greater in patients with normal hs-cTnT, suggesting that intervention may be most effective before biomarker elevation.^33^

The differing outcomes between EVOLVED and EARLY TAVR partly reflect the timing of AVR-median 5 months delay in EVOLVED vs. 14 days in EARLY TAVR. The longer delay in EVOLVED and the selection of patients with more advanced cardiac damage may have blunted the benefits of early intervention, especially since selecting patients based on myocardial fibrosis likely identifies those with already irreversible damage. In contrast, rapid intervention in EARLY TAVR preserved myocardial function and optimized clinical outcomes by treating patients before significant fibrosis develops.

In both EVOLVED^21^ and EARLY TAVR,^1^ the procedural risk associated with early intervention was low, which is consistent with the favourable safety outcomes observed in large, randomized trials of TAVR and SAVR in low-risk symptomatic patients such as PARTNER 3^34^ and Evolut Low Risk.^35^ Taken together, these data highlight that early intervention can be performed safely in selected patients and support a shift away from purely symptom-driven strategies. The primary argument for early intervention is that relying solely on symptoms risks exposing asymptomatic patients with high-gradient severe AS to acute decompensation and poorer clinical conditions at the time of AVR. Moreover, maintaining the rigorous follow-up schedules required for timely detection of symptom onset is often challenging in real-world clinical practice. Delays in access to AVR—whether due to healthcare system constraints or unexpected symptom development—can further compromise patient outcomes and increase the risk of adverse events.^31^ While the cost of TAVR and SAVR raises concerns about the justification of early intervention, particularly when follow-up is infrequent, more frequent monitoring has been proposed as a cost-effective alternative. Nonetheless, the RECOVERY and EARLY-TAVR trials indicate that early intervention may still provide substantial benefits in reducing mortality and hospitalizations, warranting further evaluation of its cost-effectiveness.

### Arguments favouring conservative watchful strategy

Despite growing enthusiasm, several considerations caution against universal early AVR in asymptomatic severe AS. Historically, asymptomatic patients with severe AS have exhibited a relatively low annual risk of sudden cardiac death, supporting the safety of a watchful waiting strategy in carefully selected individuals.^4–8^ For example, the HAVEC study^11^ reported a sudden cardiac death rate of just 0.65% per year, and a systematic review and meta-analysis by Gahl *et al*.^36^ found a sudden death rate of 1.1 per 100 patient-years (95% CI, 0.6–2.1) in asymptomatic severe AS. Additionally, a recent meta-analysis reported a cardiovascular mortality rate of 3 per 100 patient-years, while the risk of progression to symptoms or an indication for AVR was ∼18% per year.^37^

Although early intervention may offer potential advantages such as improved survival, reduced hospitalizations—including fewer hospitalizations for heart failure and lower rates of stroke as reported in recent meta-analyses—and prevention of cardiac deterioration in selected patients, the evidence for these benefits remains inconsistent, particularly in truly asymptomatic, low-risk populations.^1,19–21,31,36–38^ In fact, not all studies have demonstrated a clear and consistent mortality benefit with early intervention. The EVOLVED trial, which selectively enrolled asymptomatic patients with severe AS and myocardial fibrosis, failed to show a statistically significant reduction in the composite endpoint of all-cause mortality or unplanned heart failure hospitalization with early surgery compared to surveillance, suggesting that even advanced risk stratification may not always identify patients who will benefit from early intervention.^21^ Valve durability also remains a key consideration, particularly in younger patients with longer life expectancy; although the NOTION study^39^ has provided reassuring 10-year data with low rates of structural valve deterioration after TAVR, questions about prosthesis longevity persist as indications expand to lower-risk and younger populations.

Importantly, not all patients who develop symptoms during surveillance actually undergo AVR. In the RECOVERY trial, among patients randomized to conservative management, 40 developed symptoms or met criteria for AVR, but 16 of these (40%) did not ultimately undergo surgery.^18^ This suggests that a substantial proportion of patients may not receive timely intervention after symptom onset, either due to rapid clinical deterioration, comorbidities, or other factors. This delay between symptom onset and AVR may have contributed to the less favourable outcomes observed in the conservative arm. Similarly, in both EVOLVED^21^ and EARLY TAVR,^1^ there was a delay between meeting criteria for AVR (such as symptom onset or qualifying event) and the procedure in the conservative arms, with a median of 32 days in EARLY TAVR and a much longer delay of 20 months in EVOLVED. Such delays may have contributed to the less favourable outcomes observed with watchful waiting, as patients were at risk of progressing to advanced symptoms or adverse events before receiving intervention.

It is also crucial to recognize the heterogeneity in study populations and methodologies across major trials further complicates the interpretation and generalizability of their findings (*Table 2*).^1,18–21^ In the RECOVERY trial, AS was more severe than usual guideline criteria (mean peak velocity 5.1 ± 0.55 m/s, predominance of bicuspid valves (61%)), and the absence of symptoms was based solely on patient history without systematic exercise testing.^18^ In contrast, the AVATAR trial enrolled patients with standard definitions of severe AS and required mandatory negative exercise testing, ensuring a more rigorously defined asymptomatic cohort.^19,20^ Both trials used composite primary endpoints, but a significant reduction in all-cause mortality was observed only in RECOVERY, not in AVATAR. The patient populations were also relatively young and low-risk, and the overall sample sizes were small, limiting the robustness and applicability of the findings to the broader, often older and higher-risk, population seen in everyday clinical practice. Similarly, in EVOLVED^21^ and EARLY TAVR,^1^ differences in inclusion criteria, risk profiles, and timing of intervention further complicate direct comparisons and the extrapolation of results. For instance, EARLY TAVR required rigorous confirmation of asymptomatic status but still excluded 40% of screened patients after unmasking previously unrecognized symptoms, while EVOLVED focused on patients with myocardial fibrosis but did not demonstrate a significant mortality benefit. These methodological differences highlight the persistent challenges in accurately identifying truly asymptomatic patients and determining the optimal timing for intervention.

**Table 2:** Comparison of inclusion criteria across randomized trials of early aortic valve replacement vs. conservative management

	AVR	Age (years)	Aortic valve	Exercise testing	Surgical risks
RECOVERY	SAVR	20–80	AVA ≤ 0.75 cm^2^ and Vmax ≥ 4.5 m/s or MPG ≥ 50 mmHg	100% negative	—
AVATAR	SAVR	≥18	AVA ≤ 1.0 cm^2^ or AVAi ≤ 0.6 cm^2^/m^2^ and Vmax ≥ 4.0 m/s or MPG ≥ 40 mmHg (exclusion: Peak Vel. > 5.5 m/s)	Selective	STS < 8%
EVOLVED	SAVR/TAVR	≥18	Vmax ≥ 4.0 m/s or AVAi < 0.6 cm^2^/m^2^ and Vmax ≥ 3.5 m/s	Not required	—
EARLY TAVR	TAVR	≥65	AVA ≤ 1.0 cm^2^ or AVAi ≤ 0.6 cm^2^/m^2^ and Vmax ≥ 4.0 m/s or MPG ≥ 40 mmHg	91% negative	STS < 10%

AVA, aortic valve area; AVR, aortic valve replacement; MPG, mean aortic pressure gradient; Vmax, peak aortic velocity.

Finally, procedural risks—including stroke, bleeding, and especially the need for permanent pacemaker implantation—persist despite technological advances.^1,21,34,35^ Systematic early intervention would expose many patients, who might otherwise remain stable for years, to these risks unnecessarily. From a healthcare system perspective, universal early intervention would significantly increase procedural volume and resource utilization, without proven benefit for all.

The identical mortality rates observed in PARTNER 3,^34^ Evolut Low Risk,^35^ EVOLVED,^21^ and EARLY TAVR^1^ trials demonstrate that, in low-risk or selected asymptomatic patients, a strategy of watchful waiting with intervention at symptom onset offers equivalent safety to systematic early intervention, at least in terms of medium-term survival. Moreover, as highlighted in the recent review by Genereux, much of the evidence supporting early TAVR derives from observational studies rather than large, randomized trials, introducing potential biases that may overestimate benefits.^31^

In summary, while early intervention may benefit selected high-risk individuals, the evidence for a consistent mortality benefit remains limited. For most truly asymptomatic, low-risk patients, a conservative watchful waiting strategy with structured follow-up remains appropriate and aligned with current guidelines, given the low annual risk of sudden death, lack of uniform mortality benefit, concerns over valve durability, and procedural risks.

### Integrating expert opinion with clinical guidelines

Current international guidelines (ACC/AHA 2020, ESC 2021) recommend conservative management for asymptomatic severe AS, reserving intervention for those with established high-risk features such as reduced LV ejection fraction (<50%), abnormal exercise testing, or rapid haemodynamic progression.^15,16^ However, recent prospective studies, including the EARLY TAVR trial, have highlighted significant limitations of this approach.^21,22^ Notably, a substantial proportion of patients initially classified as asymptomatic are reclassified as symptomatic after systematic evaluation, and up to 47% of patients under surveillance develop symptoms or adverse events within 1 year.^22^ This rapid progression suggests that the traditional 6–12-month follow-up interval may be insufficient for timely detection of clinical deterioration, particularly in patients with high-gradient AS or borderline findings.^17^

**Figure 3:**
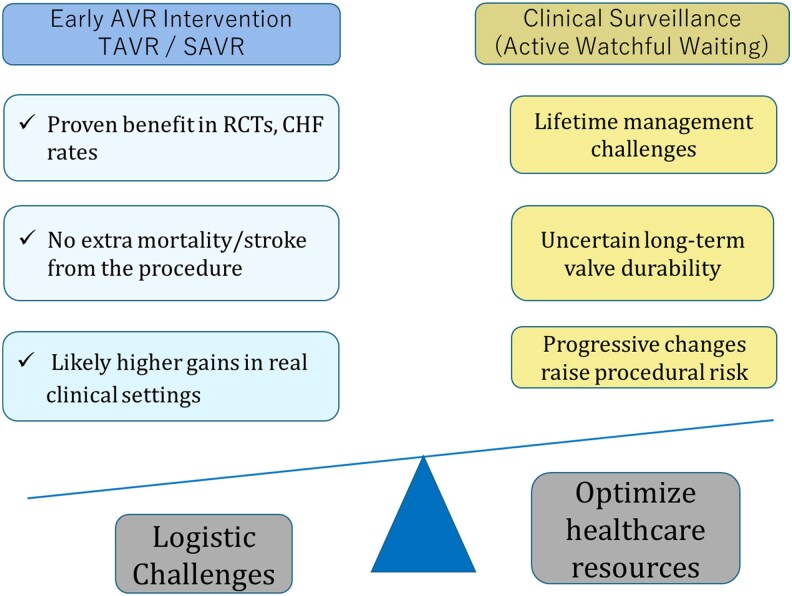
Balancing early aortic valve intervention vs. clinical surveillance in asymptomatic severe AS. This schematic compares the benefits and challenges of early aortic valve replacement (AVR)—including TAVR and SAVR—vs. an active surveillance strategy in asymptomatic patients with severe aortic stenosis. While early intervention demonstrates favourable outcomes in randomized trials and may offer greater gains in real-world practice without added procedural risk, clinical surveillance raises concerns related to valve durability, patient progression: transition from asymptomatic to symptomatic AS or progression to advanced cardiac damage stages, and timing of intervention. System-level factors such as resource optimization and logistical capacity also weigh into the treatment decision.

This FDA approval, based on EARLY TAVR,^1^ highlights logistical challenges such as increased procedural volume and the need for judicious patient selection to avoid overtreatment and optimize healthcare resources (*Figure 3*).

**Figure 4:**
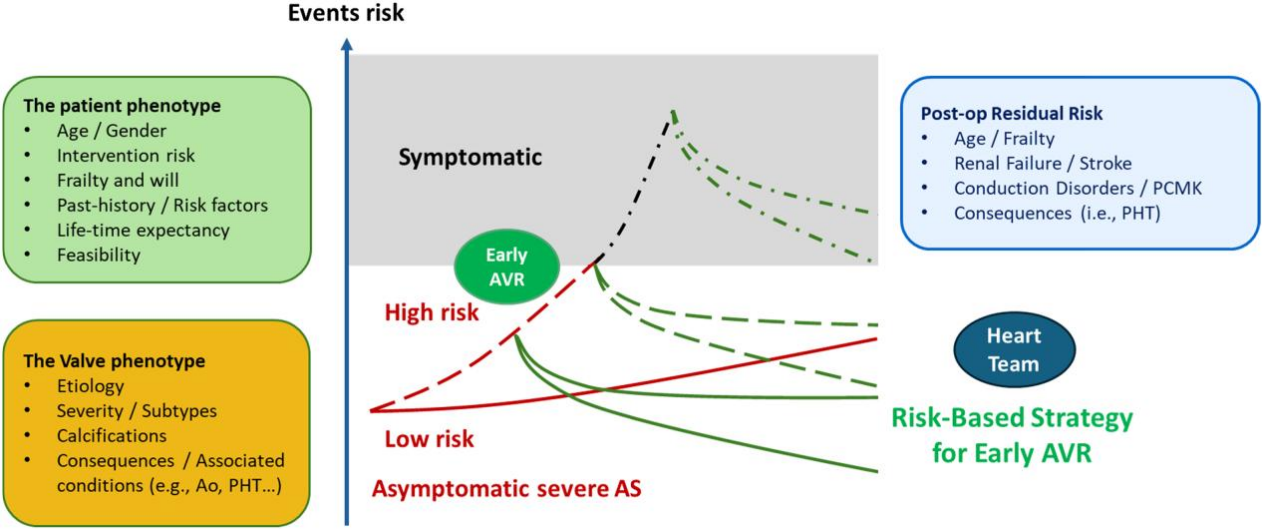
Prognostic divergence according to patient and valve phenotypes in severe aortic stenosis. In asymptomatic severe aortic stenosis, prognosis diverges over time based on baseline patient characteristics (age, frailty, comorbidities) and valve pathology (stenosis severity, calcification burden, remodelling). Both with and without aortic valve replacement (AVR), residual patient-related risks—including frailty, stroke, renal failure, and conduction disorders—critically influence long-term outcomes. Ao, aorta; AVR, aortic valve replacement; PCMK, pacemaker; PHT, pulmonary hypertension.

Given the heterogeneity of both patient and valve phenotypes—including age, frailty, comorbidities, life expectancy, degree of valve calcification, and patterns of ventricular remodelling—disease trajectory and risk of adverse outcomes can vary widely (*Figure 4*). Emerging evidence suggests that early markers of myocardial dysfunction, subclinical imaging abnormalities, or high-gradient haemodynamics may identify patients at higher risk of rapid progression to symptomatic AS or irreversible myocardial damage, even in the absence of overt symptoms.^4–15^ Accordingly, expert consensus now advocates for a more personalized and adaptive surveillance strategy—one that incorporates patient preferences, advanced multimodal imaging, biomarker evaluation—and comprehensive clinical profiling, to enhance risk stratification and optimize the timing of intervention.

**Figure 5:**
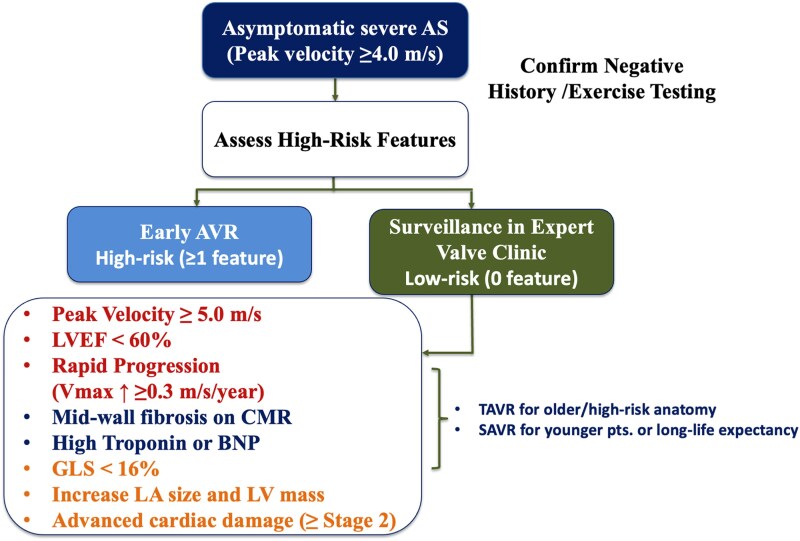
Risk-based strategy for early AVR in asymptomatic severe AS. Suggested clinical algorithm for managing asymptomatic patients with severe AS (peak velocity ≥ 4.0 m/s). High-risk features such as elevated biomarkers, reduced LVEF, myocardial fibrosis, and impaired GLS may warrant early AVR. TAVR is preferred in older or high-risk patients, while SAVR is recommended in younger individuals with longer life expectancy. BNP, B-type natriuretic peptide; CMR, cardiac magnetic resonance; GLS, global longitudinal strain; LA, left atrium; LVEF, left ventricular ejection fraction.

Contemporary risk assessment should incorporate not only symptoms and valve haemodynamics but also markers such as peak aortic velocity ≥ 5.0 m/s, declining or borderline LVEF (<60%), impaired global longitudinal strain (GLS > −16%), elevated biomarkers (high-sensitivity troponin, BNP), and evidence of adverse remodelling (increased LV mass index, left atrial enlargement) or of advanced cardiac damage (≥Stage 2) (*Table 3*) (*Figure 5*). Borderline or declining LVEF should be confirmed by repeat measurement within 2–3 weeks; if uncertainty persists, advanced imaging modalities such as 3D echocardiography or cardiac magnetic resonance (CMR) should be employed for greater accuracy and tissue characterization.^40^ CMR can detect mid-wall fibrosis and extracellular volume expansion, which are associated with adverse outcomes. However, the EVOLVED trial^21^ showed no significant overall benefit from early intervention in patients with myocardial fibrosis, highlighting that its role in guiding systematic intervention remains to be fully established. Nevertheless, given its association with adverse prognosis, the presence of mid-wall fibrosis should be taken into account in the overall risk assessment and may support consideration of earlier intervention as part of an individualized treatment strategy.

**Table 3:** Expanded risk stratification model for early AVR

Risk marker	Guidelines 2020/2021	Proposed expansion	Risk category
Classic high-risk markers			
Very severe AS (Vmax ≥ 5.0 m/s)	(IIa-B)^a,b^	Yes	Immediate event risk
LVEF < 50%	(I-B)^a,b^	Yes	Severe LV systolic dysfunction
Positive stress test	(I-B)^a,b^	Yes	Unmasked symptomatic AS
Rapid progression (Vmax ↑ ≥ 0.3 m/s/year)	(IIa-B)^c,d^	Yes	High-risk disease evolution
Emerging cardiac damage markers			
LVEF 55–60%	No (IIa-B for 55^b^**^—^**60%^a^)	Yes	Early LV dysfunction
Impaired GLS (<−16%)	No	Yes	Early systolic dysfunction
Fibrosis on CMR	No	Yes	Irreversible myocardial injury
Elevated troponin (≥6 ng/L)/BNP	No	Yes	Subclinical myocardial stress
Left atrial enlargement (volume > 34 mL/m²) and increased LV mass index	No	±	Diastolic dysfunction; risk of AF/HF—chronic pressure overload

Proposed expansion: Risk markers suggested as potential indications for early AVR, beyond those currently recommended in the ACC/AHA 2020 and ESC 2021 guidelines.

AS, aortic stenosis; AVR, aortic valve replacement; CMR, cardiac magnetic resonance imaging; GLS, global longitudinal strain; LVEF, left ventricular ejection fraction; BNP, B-type natriuretic peptide.

^a^ACC/AHA guidelines.

^b^ESC guidelines.

^c^If high gradient.

^d^If severe calcification and low risk.

Integrating comorbidities, particularly heart failure with preserved ejection fraction (HFpEF), is essential, as they may significantly contribute to cardiac damage, myocardial fibrosis, and symptoms that are not directly attributable to AS.^41^ These conditions warrant targeted medical therapy, including the use of SGLT2 inhibitors, both before and after valve intervention.^42^

Given these considerations, a more intensive follow-up schedule—every 3 months rather than the traditional 6–12 months—may be warranted, especially for patients with high-gradient AS, borderline symptoms, or early evidence of cardiac remodelling. This approach aims to capture early clinical or subclinical deterioration, facilitate timely referral for valve intervention, and ultimately prevent irreversible myocardial damage. Procedural strategy should also be individualized: TAVR is preferred for elderly or higher-risk patients with favourable anatomy, while surgical AVR remains the standard for younger or lower-risk individuals due to concerns regarding long-term valve durability.^43^ However, procedural risk must also be considered separately from the risk of clinical deterioration during watchful waiting. Low procedural risk—particularly in patients eligible for transfemoral access with minimal anatomical complexity—may favour early intervention. In contrast, patients with high procedural risk, such as those with severely calcified valves extending into the LVOT, low-lying coronary ostia, narrow sinuses of Valsalva, or requiring upper-body access, may not be ideal candidates for an early invasive strategy, regardless of symptom status. Ultimately, all management decisions should be made within a multidisciplinary heart team, integrating clinical, imaging, and biomarker data to optimize intervention timing according to patient risk profile and preferences. It is also important to note that the benefits of early TAVR require rapid intervention, often within a month, which may not be feasible in everyday practice.^1^ In this new era, the challenge will be to implement a precision medicine approach, ensuring that early intervention is targeted to those with the highest risk phenotype and most likely to benefit, while maintaining system sustainability and patient safety. However, the current evidence remains limited by small sample sizes, heterogeneous patient populations, and inconsistent outcomes, underscoring the need for further large, randomized controlled trials. The ongoing EASY-AS trial, which is evaluating early SAVR in asymptomatic severe AS, is expected to provide important insights to help refine management strategies and warrants close attention.

## Conclusion

Early aortic valve replacement (AVR) in asymptomatic severe AS is now a central question in valve management. Recent trials and the FDA approval of TAVR for asymptomatic patients support a proactive approach, showing benefits in reducing major cardiovascular events and preventing irreversible myocardial damage. However, the selection process of the timing of intervention should also weigh in the procedural risks and the proven durability of the bioprosthetic valve. Prognosis varies early according to patient and valve phenotype, limiting the value of a symptom-driven strategy alone. Multimodal, individualized risk stratification—using imaging, biomarkers, and clinical assessment—should guide selection for early AVR, reserving expert surveillance for lower-risk profiles. This patient-centred, phenotype-driven approach is essential as indications expand, and more candidates are considered for intervention.

## Data Availability

There are no new data associated with this article.
